# Droplet Controlled Growth Dynamics in Molecular Beam Epitaxy of Nitride Semiconductors

**DOI:** 10.1038/s41598-018-28984-9

**Published:** 2018-07-26

**Authors:** Mani Azadmand, Luca Barabani, Sergio Bietti, Daniel Chrastina, Emiliano Bonera, Maurizio Acciarri, Alexey Fedorov, Shiro Tsukamoto, Richard Nötzel, Stefano Sanguinetti

**Affiliations:** 1 L-NESS and Dipartimento di Scienza dei Materiali, Università di Milano-Bicocca, Milano, Italy; 2 L-NESS and Dipartimento di Fisica, Politecnico di Milano, Como, Italy; 3 L-NESS and IFN–CNR, Milano, Italy; 4 South China Academy of Advanced Optoelectronics, South China Normal University, Guangzhou, China

## Abstract

The growth dynamics of Ga(In)N semiconductors by Plasma-Assisted Molecular Beam Epitaxy (PAMBE) at low temperatures (*T* = 450 °C) is here investigated. The presence of droplets at the growth surface strongly affects the adatom incorporation dynamics, making the growth rate a decreasing function of the metal flux impinging on the surface. We explain this phenomenon via a model that considers droplet effects on the incorporation of metal adatoms into the crystal. A relevant role is played by the vapor-liquid-solid growth mode that takes place under the droplets due to nitrogen molecules directly impinging on the droplets. The role of droplets in the growth dynamics here observed and modeled in the case of Nitride semiconductors is general and it can be extended to describe the growth of the material class of binary compounds when droplets are present on the surface.

## Introduction

Among compound semiconductors, InGaN has very unique properties such as high near band edge absorption, high carrier mobility, surface electron accumulation, and superior radiation resistance^1–4^. But what puts this material in high demand for many industrial applications is its wide tunability of the band gap which spans from 0.7 to 3.4 eV, depending on the In composition^5^. This large energy range covers almost the whole solar spectrum thus making InGaN the optimal material for solar applications^6^. InGaN characteristics are desirable for many applications such as light sources, light detectors, solar cells, photo electrochemical water splitting and electrochemical biosensors^7–9^. Moreover, growth of low to high indium composition InGaN directly on Si has recently been demonstrated^10^ for cost reduction and integration with Si technology. InGaN growth is hindered by the lattice mismatch and the different thermal stabilities of the two bond types present in the material: In-N and Ga-N. The lattice mismatch leads to a miscibility gap which can cause fluctuations of the In content in the epilayer^11,12^.

The different binding energies of In-N and Ga-N bonds are reflected in the different decomposition temperatures of InN (630 °C) and GaN (850 °C)^13^. Consequently, a reduction of In incorporation in the epilayer occurs not only due to the re-evaporation of adsorbed surface adatoms but also due to the thermal decomposition of In-N bonds. So far, many works have been devoted to the study phenomena such as phase separation^14^, surface reconstruction during transition between metal-rich and N-rich regions^15^, and the other growth behaviors of InGaN grown mostly at high (normal) temperatures^16–21^. Low growth temperatures have been used to avoid InN decomposition and In desorption, thus allowing the growth of high-In-composition InGaN layers by plasma-assisted molecular beam epitaxy (PAMBE)^10,22–24^. At these temperatures, metal-rich conditions easily lead to the formation of droplets on the surface which affect the film quality^25^ and that are difficult to remove by thermal treatment, because the In-N dissociation temperature is lower than the onset temperature for the evaporation of In from the In droplets on the InGaN surface^26^. This has been shown to be detrimental to material properties and a major drawback for device applications^27^ although there was no clear explanation reported for these phenomena. At higher substrate temperatures, an intermediate Ga-rich regime is observed, leading to smooth droplet-free surfaces^28^. Several method to overcome such drawback have been proposed, based on the alternating sequence of metal-rich and nitrogen-rich conditions at low temperature to take profit of the higher mobility of metal adatoms in metal-rich conditions and to eliminate the metal droplets that form on the surface during the atomic nitrogen irradiation in absence of metal flux^23,29,30^.

However, there is still little knowledge about the exact growth dynamics of InGaN in the low temperature regime and the role that is played by the metal droplets that form at the surface in the metal-rich conditions. In this study we demonstrate that the droplets have a fundamental role in determining the InGaN growth dynamics determining the adatoms kinetics of incorporation in the presence of droplets. We propose a theoretical approach that models the observed phenomena highlighting the role of the droplet as a sink for the metal adatoms and the role of the Vapor Liquid Solid (VLS) growth mode that takes place under the droplets in the InGaN epilayer growth.

## Experimental

The InGaN thin-films were grown by molecular beam epitaxy (MBE) equipped with a radio frequency (RF) plasma source for nitrogen, on Si (111) substrates. The native silicon oxide was removed from the surface by a thermal annealing at 850 °C for 30 min in vacuum. Prior to the growth, the substrates were exposed to the nitrogen plasma with a flux of 0.9 sccm (standard cubic centimeter per minute) and RF power of 360 W at 800 °C. This procedure has been established to grow high quality GaN and InGaN epilayers on Si^7,31^. Subsequently the temperature of the sample was reduced to 450 °C (growth temperature) and the InGaN thin film was grown with equal Ga and In fluxes (see Table 1). The same N flux and RF power was maintained as during the nitridation step.

**Table 1:** Metal flux (In +Ga with one to one ratio), growth time, and growth rate of samples.

Sample	Metal flux (10^14^ atoms cm^−2^ s^−1^)	Growth time (mins)	Metal dose (10^16^ atoms cm^−2^)	Growth rate (nm/min)
A	0.39	120	28	0.65
B	0.78	120	56	1.20
C	0.98	120	71	1.67
D	1.17	120	84	1.46
E	1.41	90	76	1.30
F	1.96	45	53	1.37
G	2.94	45	79	1.32
H	2.94	90	158	1.42
I	2.94	150	264	1.67
J	3.92	60	141	1.03
K	7.83	90	423	0.83

After growth, the samples were rapidly cooled down to room temperature and taken out of the MBE chamber for further characterization.

X-ray diffraction (XRD) was employed to examine the sample composition. The surface morphology was investigated by both optical microscopy as well as scanning electron microscopy (SEM) using a secondary electron detector. The layer thickness, and in turn the growth rate, was determined via cross-section SEM images of the samples.

## Results and Discussion

**Figure 1:**
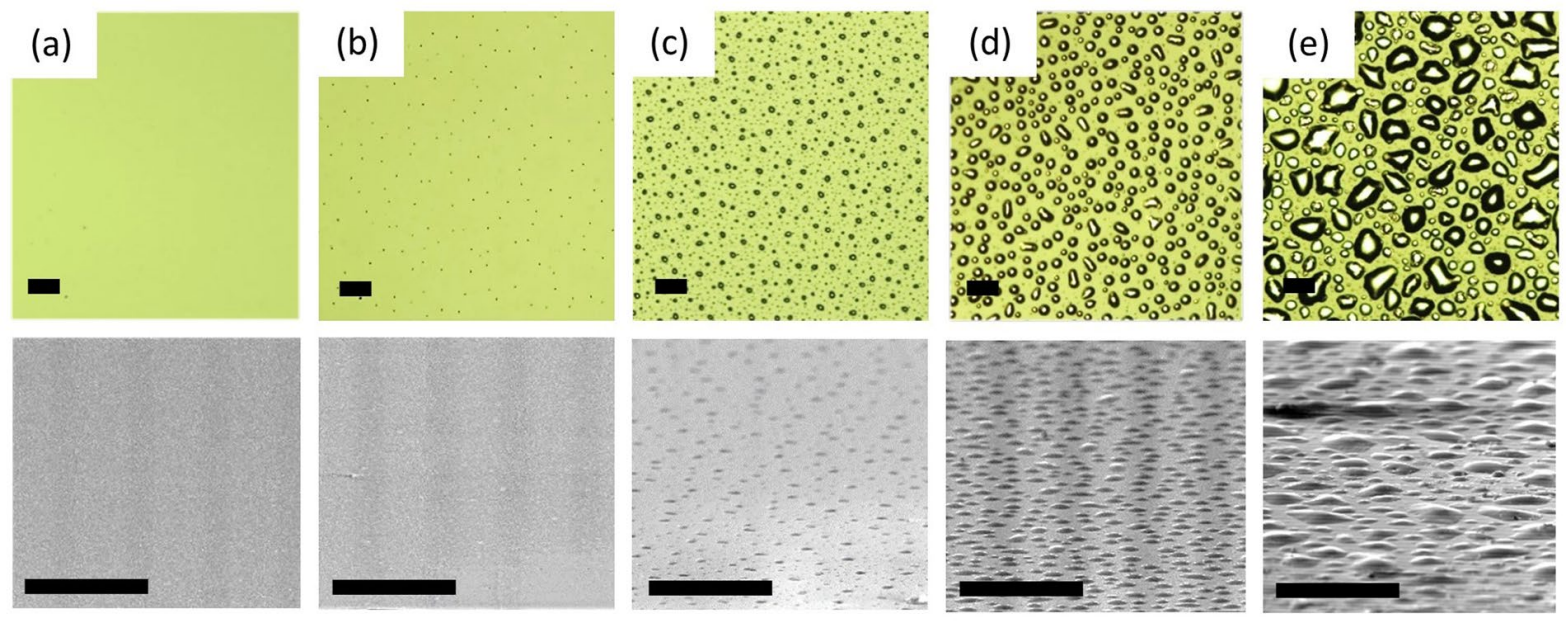
(Top) Optical Microscope and (bottom) related SEM images (taken at an angle of 70° respect to the sample) of InGaN samples grown under different metal fluxes, from left to right: (**a**) 0.39 × 10^14^, (**b**) 0.98 × 10^14^, (**c**) 1.41 × 10^14^, (**d**) 3.92 × 10^14^, (**e**) 7.83 × 10^14^ atoms cm^−2^ s^−1^. Black line length in all the images is 100 μm.

**Figure 2:**
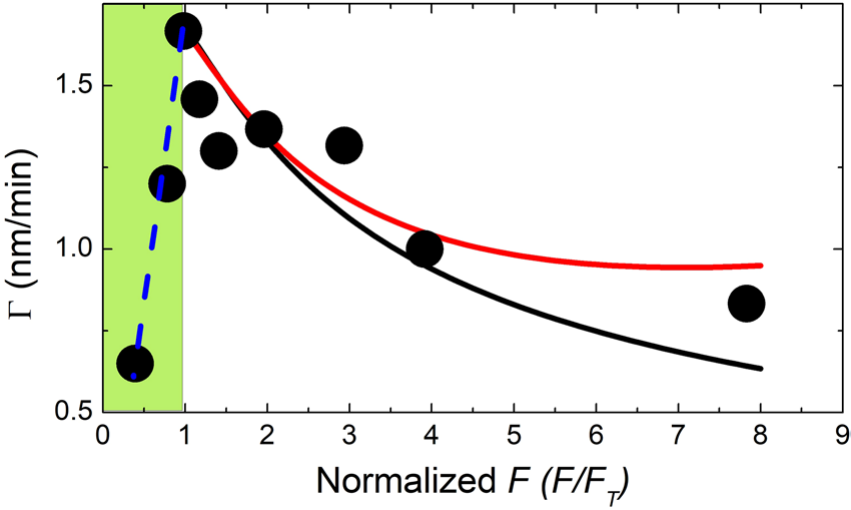
Average growth rate as a function of metal flux *F* normalized by *F*_*T*_ = 0.98 × 10^14^ atoms cm^−2^ s^−1^. The dashed lines correspond to the model predictions: Initial linear dependence in absence of droplets (blue), Г (red) and R_S_ (black). Model fit parameters are *R*_*U*_/*R*_*S*_ (*F*_*T*_) = 0.9 ± 0.2, *σρ*_*0*_/*δ* = 3 ± 1, the critical exponent *p* = 1.6 ± 0.1. The size of the experimental points indicates the error of the data.

Figure 1(a–e) show the optical microscopy and SEM images taken from samples with different metal fluxes. Epilayer surfaces are smooth and free of metal droplets when the metal flux is not too high, that is, when the growth presumably takes place in slightly N-rich conditions (excess of active N atoms compared to metal atoms reaching the surface). In this regime the growth rate is proportional to the impinging metal flux (green shaded area in Fig. 2). The droplets start to appear as the ratio of metal flux vs nitrogen flux exceeds the equilibrium point, which in our experiments is *F*_*T*_ = 0.98 × 10^14^ atoms cm^−2^ s^−1^. This happens when two Ga atoms and/or more form a stable initial cluster on the surface. The same kinds of process for the Ga clusters, starting with two Ga atoms, were previously observed by *in-situ* scanning tunneling microscopy (STM) during a MBE growth on GaAs(001) substrate^32^. The creation of metal droplets in the metal-rich conditions has been already reported in case of GaN and in general in the growth of InGaN grown at low temperature^23–26,29,33–35^. Here we observed that as the droplets start to appear on the surface, a marked decrease in growth rate (see Fig. 2) occurs. The growth rate depends strongly on the metal flux, decreasing as the flux increases, and this effect starts as soon as droplets are formed on the surface (Fig. 2). At the end of the growth, for *F* > *F*_*T*_, we observe surface covered by metal droplets. It is worth mentioning that the total amount of metal stored in the droplets corresponds, within 20% accuracy, to the total dose of metal deposited, once the fraction incorporated in the InGaN crystal has been subtracted. However, it is found that in presence of droplets, by increasing the time of growth (at the same metal/N ratio), the growth rate increases (see samples G,H and I in Table I).

To understand the observed behavior, we carefully consider the metal adatom dynamics during the growth in the transition between the N-rich and the metal-rich regimes. Among all growth parameters, the III/N ratio is one of the key points to improve the surface structure and morphology of the grown film. Based on this ratio, two different morphologies have been observed. One is the smooth epilayer structure which is achieved in metal-rich and lightly N-rich conditions and the other is the so-called nano-columnar structure which will appear in case of N-rich growth^36^. Nitride semiconductor films grown with even a slight excess of N during growth (N-stable conditions) display a rough surface morphology with a columnar structure initiated by the formation of stacking faults^37^. This can be explained considering the very different diffusivity for Ga and N adatoms on the surface. While Ga is very mobile at typical growth temperatures, the diffusion of N is slower by orders of magnitude. The presence of excess N strongly increases the Ga diffusion barrier. It has been calculated that the diffusion barrier for Ga adatoms on N-rich surfaces is as high as 1.8 eV, whereas it is only 0.4 eV when the growth is carried out on a Ga-saturated surface. Thus, N-rich growth leads to very low adatom mobility and to an undesired kinetically induced roughening of the surface^38^. GaN growth by PAMBE is commonly carried out under metal-rich conditions. But it must not be forgotten that in metal-rich regimes where there is the accumulation of metal atoms and creation of droplets on the surface, these very mobile metal adatoms on the surface can join metal droplets instead of participating in the crystal growth.

**Figure 3:**
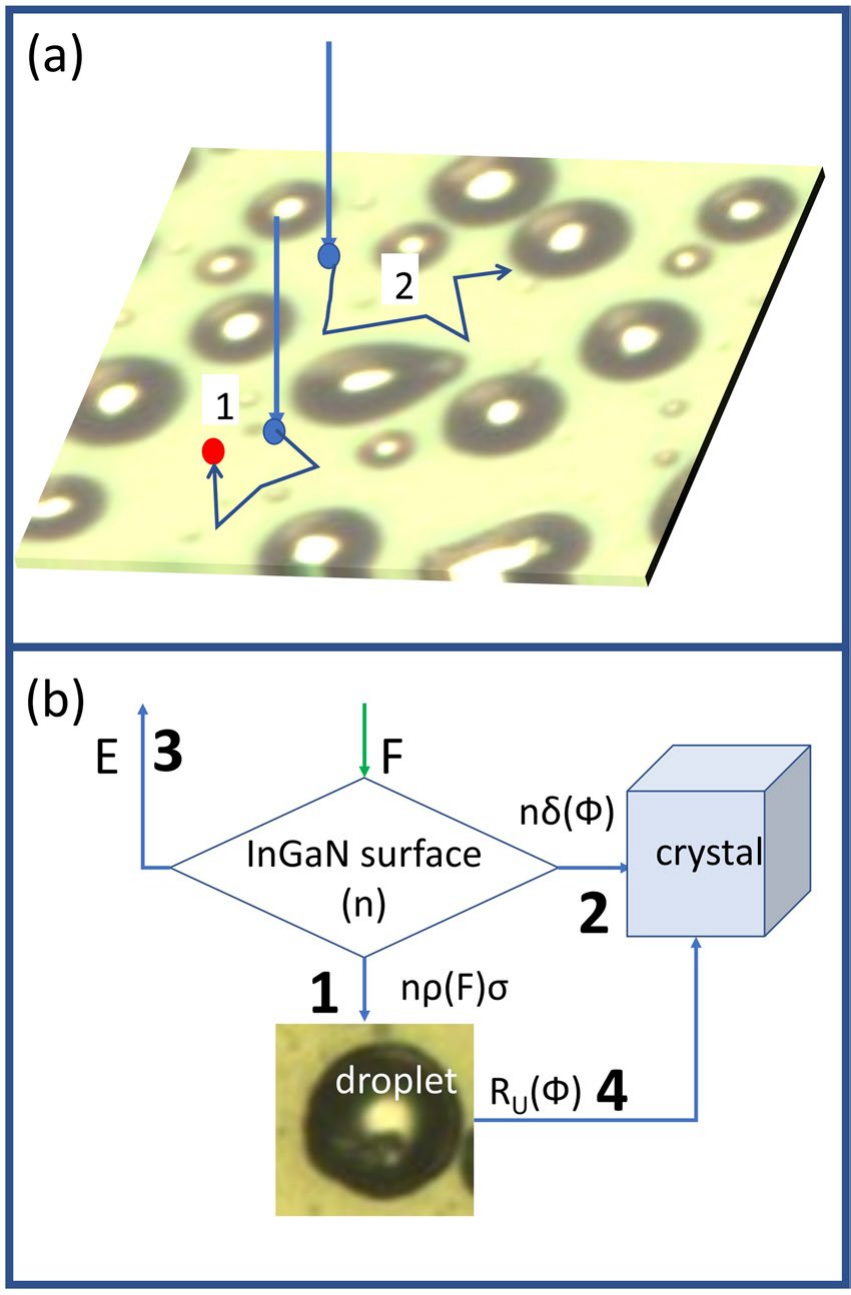
(**a**) Available metal adatom processes. Process 1 leads to metal droplet attachment. Process 2 leads to incorporation into the growing crystal (indicated by the red dot). (**b**) Schematics of the metal adatom rate equation model highlighting the four available channels for the metal adatoms: (1) droplet attachment; (2) crystal incorporation; (3) desorption; (4) VLS crystal growth under the droplet.

We observe that the decrease in the growth rate is always accompanied by the presence of metal droplets on the sample surface after the growth. The growth rate decreases more and more with the increase of the metal flux and so does the density and volume of droplets, though the N flux is unchanged. This is in marked contrast to the common assumption that the growth rate under metal rich conditions is determined by the N flux. Hence, the common N flux calibration procedure under metal-rich conditions must be reconsidered. The droplets thus should play a fundamental role in the change of metal adatom incorporation dynamics. As soon as the number of metal atoms exceeds the critical density for droplet formation, the metal atoms start to form droplets and accumulate in them. The presence of droplets on the surface establishes a depletion channel for the metal adatom density on the growth surface, as metal adatoms can be efficiently captured by the droplets. This depletion channel is in competition with the N-driven metal adatom incorporation into the InGaN crystal. This leads, on one hand, to a reduction of the metal adatom incorporation rate into the crystal in the regions not covered by the droplets. On the other hand, the presence of liquid metal droplets on the surface should allow for the VLS growth of InGaN material under the droplets themselves, via direct incorporation of nitrogen. In summary, three possible processes are available to the metal adatom on the growing InGaN surface in the presence of droplets (see Fig. 3):

1. Incorporation of metal adatom into the crystal by binding to a N active site on the droplet-free surface.
2. Metal adatom attachment to a droplet.
3. Desorption.

In addition, the metal adatoms incorporated in the droplets can be incorporated in the crystal due to VLS mechanism under the droplet by direct N flux into the droplet (process 4). It is possible then to model the combined effect of the three processes as a set of rate equations for the metal adatom density *n* and VLS growth rate (see b). The first equation describes the kinetics of the metal adatom density *n*, determined by the combined effect of external metal flux, incorporation in to the crystal and attachment to the droplets:

1 $$\frac{{\boldsymbol{dn}}}{{\boldsymbol{dt}}}={\boldsymbol{F}}-{\boldsymbol{E}}-n{\boldsymbol{\delta }}({\boldsymbol{\Phi }}/{\bf{F}})-n{\boldsymbol{\sigma }}{\boldsymbol{\varrho }}({\boldsymbol{F}})$$

where *F* is the total (In plus Ga) metal flux, *Φ* is the active N flux, *E* is the desorption flux, *δ(Φ/F)* the incorporation probability into the crystal on the droplet free surface, which depends on the ratio between active N flux *Φ* and the metal flux *F*. *δ(Φ/F)* is proportional to the probability, for a metal adatom, to find an active N site for binding. *ρ(F)* the droplet density and σ the droplet capture cross section. We cannot access experimentally the exact density of droplets acting during the growth. The droplet density that is measured after the growth, especially at high coverage, it is affected by Ostwald ripening effects that take place during the cooling of the sample and it is therefore not clearly related to the droplet density in the growth regime. Therefore the droplet density and its dependence on the metal flux will be treated as a model parameter. At low temperature in MBE conditions the metal desorption flux is close to zero (*E* = 0). Therefore in the steady state condition, Eq. (1) leads to the solution for *n*

2 $$n=F\frac{1}{\delta ({\rm{\Phi }}/F)+\sigma \rho }$$

And consequently, the bulk growth rate per unit surface area (process 2) *R*_*S*_ that takes place in the regions of the sample where there are no droplets is:

3 $${R}_{S}(F,{\rm{\Phi }})=n\delta ({\rm{\Phi }}/F)=F\frac{1}{1+\frac{\sigma {{\varrho }}_{o}{F}^{p}}{\delta ({\rm{\Phi }}\,/\,F)}}$$

here we explicitly used the expected dependence of the droplet density on the metal flux *ρ(F)* = *ρ*_0_*F*^*p*^^39^. *p* is the critical exponent for droplet nucleation, whose value depends on physical processes responsible for the formation of the droplets: (i) the size of the critical nucleus in terms of atoms^32^, (ii) the probability of metal atoms from the flux to stick on the surface and to be incorporated in the adatom population. The latter process is usually classified in “complete condensation” and “incomplete condensation” regimes. These term, in the theory of nucleation of stable adatom clusters on the growth surface, refers to a condition where the actual flux of adatom from the gas is fully adsorbed (complete condensation) or not-fully adsorbed (incomplete condensation) due to desorption or other leaky channels^39,40^. As droplet formation during growth of happens in the presence of a leak channel for the adatoms, that is the bulk incorporation, we expect to be in the regime of incomplete condensation. In this regime, *p* > 1 values are expected^39^.

It is worth mentioning that in the N-rich region, where the density of droplets is equal to zero (green region in Fig. 2), Eq. (3) reduces to:

$${R}_{S}(F,{\rm{\Phi }})=F$$

In N-rich conditions the predicted InGaN growth rate linearly follows the metal flux, as observed. When the growth turns to metal-rich conditions, as soon as the adatom density *n* overcomes the critical density for droplet formation, the second depletion channel opens for the metal adatoms (in addition to the incorporation), thus leading to a rapid depletion of the adatom density *n*. The depletion of *n* leads a reduction of *R*_*S*_ in the area between the droplets. The effect becomes larger as the metal flux increases, as the droplet density, and thus in turn the metal adatom capture probability by the droplets, increases with *F*. The transition between the two growth modes takes place around *F*_*T*_ = 0.98 × 10^14^ atoms cm^−2^ s^−1^.

**Figure 4:**
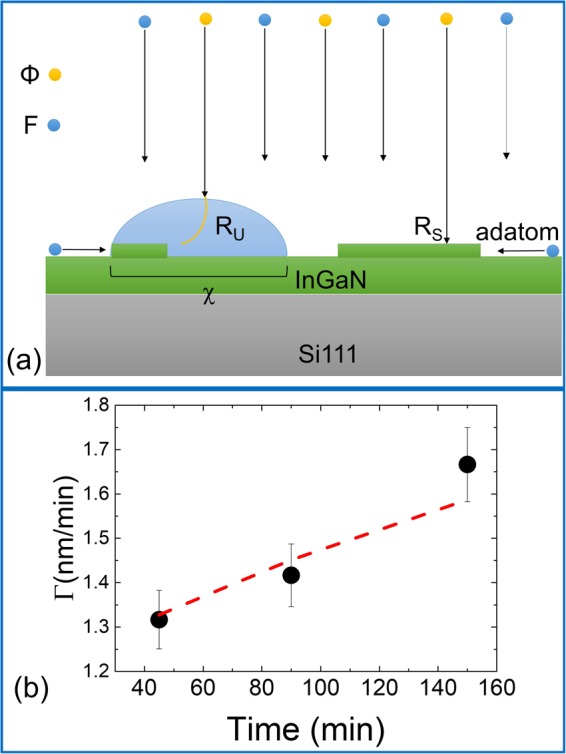
(**a**) Schematic of crystal growth under and between the metal droplets. (**b**) Observed dependence of average growth rate from growth time (black dots). The dashed line represents the model predictions [Eq. (7)]. Here *R*_*C*_ = 67 nm/h and 3*γB*/5 = 15 nm/h^1/3^. Metal flux used in the growth: *F* = 2.94 × 10^14^ atoms cm^−2^ s^−1^.

However, under the droplets, VLS growth can take place, due to the incorporation of nitrogen molecules into the droplets by direct impingement^41^. An additional nitrogen incorporation mechanism in to the droplet, with subsequent VLS growth at the basis of the droplet, has been identified by means of molecular dynamics simulations by Kawamura *et al*.^42^ and related to N migration on stable InGaN surfaces. The growth rate per unit area under the droplet is *R*_*U*_*(Φ*) and depends on the nitrogen flux *Φ*, the incorporation probability of N into the droplet and the growth rate at the liquid-solid interface. The total growth rate is therefore the sum of the two contributions, that is the growth rate *R*_*S*_ in the droplet free areas and *R*_*U*_ under the droplets, whose relevance is given by the surface area covered by the droplets (see Fig. 4). The total growth rate per unit area is then:

4 $${R}_{T}(F,{\rm{\Phi }},\,{\rm{\tau }})=(1-\chi ){R}_{S}+\chi {R}_{U}={R}_{S}+\chi ({R}_{U}-{R}_{S})$$

where *χ* is the relative area covered by the droplets. This depends on the total amount of metal stored in the droplet ensemble and on the droplet density *ρ*. As long as the droplets do not touch (*χ* ≪ 1), the dependence of *χ* on the metal flux *F*, the deposition time *τ* and the droplet density *ρ* is:

5 $$\chi (\tau )=\gamma {[\frac{(F-{R}_{S})\tau }{\rho }]}^{2/3}\,\rho =\gamma {{\varrho }}_{0}^{1/3}{(1-\frac{1}{1+\frac{\sigma {{\varrho }}_{o}{F}^{p}}{\delta (\frac{{\rm{\Phi }}}{F})}})}^{2/3}{F}^{\frac{2+p}{3}}{\tau }^{2/3}$$

where γ is a normalization constant which can be derived from experiments by equating the Eq. (5) predictions with the observed *χ* at maximum metal flux. (*F*−*R*_*S*_)*τ* is the total metal quantity that is stored in the droplet ensemble after the growth time τ. It corresponds to the total metal dose deposited on the substrate minus the amount incorporated in the crystal between the droplets (*R*_*S*_). We did not consider the decrease in the metal dose available for droplet formation due to the crystallization under the droplet as it is a small correction for *χ* ≪ 1 and it adds complexity to the model. It is worth mentioning that Eq. (5) is not valid for long growth times, when *χ* → 1 and the amount of growth proceeding via VLS under the droplets becomes relevant. By combining equations (3–5) we find the dependence of the total growth rate, at time τ, per unit area to be given by the relation

6 $${R}_{T}=F\,\frac{1}{1+\frac{\sigma {{\varrho }}_{o}{F}^{p}}{\delta ({\rm{\Phi }}\,/\,{\rm{F}})}}+\gamma [{{\varrho }}_{0}^{1/3}\,({R}_{U}({\rm{\Phi }})-F\,\frac{1}{1+\frac{\sigma {{\varrho }}_{o}{F}^{p}}{\delta ({\rm{\Phi }}\,/\,F)}}){(1-\frac{1}{1+\frac{\sigma {{\varrho }}_{o}{F}^{p}}{\delta ({\rm{\Phi }}/F)}})}^{2/3}{F}^{\frac{2+p}{3}}]{\tau }^{2/3}$$

The total growth rate *R*_*T*_ *versus* time dependence is introduced by the change, with the growth time, of the area covered by the droplets. The exact τ^2/3^ dependence is related to the invariance of the wetting angle of the droplet with its size. In the extreme case of a surface covered by closely arranged droplets, the growth rate will be determined by the N flux only, that is *R*_*T*_ ∝ Φ. It is worth mentioning that the growth progressively shifts towards the VLS dominated mode as the growth time increases in metal-rich conditions.

From equation (6) the dependence on time of the average growth rate Г is

7 $$\,{\rm{\Gamma }}={R}_{S}+\frac{3\gamma }{5}B(F,{\rm{\Phi }}){\tau }^{2/3}$$

where *B(F*, *Φ)* indicates the complex formula encased in the square brackets in Eq. (6).

At first glance, the parameters of the model that enter Eq. (6) are quite numerous, which would make the extraction of the parameter values rather difficult. However, it is possible to reduce consistently the number of free parameters through the following considerations. The parameter *γ*, the prefactor of the droplet coverage dependence, can be extracted from Eq. (5) once the surface coverage for a sample is known. We used as reference the sample K, the one obtained at maximum metal flux, where χ = 0.6. The model scaling factor can be adjusted by setting *Г(F*_*T*_*)* = *Г*_*MAX*_ = 1.7 nm/min. The parameters *σ, ρ*_0_ and *δ* are always present in the form of the ratio *σρ*_0_*/δ*. Therefore, the only free parameters to be found by fitting procedures are then: i) the ratio *R*_*U*_*/R*_*S*_, ii) the ratio *σρ*_0_*/δ* and iii) the critical exponent *p*. The fitted values are: *R*_*U*_*/R*_*S*_ *(F*_*T*_) = 0.9 ± 0.2*, σρ0/δ* = 3 ± 1 and the critical exponent *p* = 1.6 ± 0.1. The dependence of the growth rate on the metal flux is reported in Fig. 2. After the initial linear dependence on *F*, the model curve shows a clear decrease in the growth rate. The good agreement of the model based on the dependence of *R*_*S*_ on *F* (black curve) indicates that the main factor determining the reduction of the growth rate is the decrease in the adatom density *n* caused by the opening of the droplet depletion channel. The critical exponent extracted from the model is *p* = 1.6 ± 0.1, thus showing that the droplet density is increasing superlinearly with the increasing metal flux as expected being the droplet nucleation in the regime of incomplete condensation, since in presence of nitrogen, a fraction of the deposited metal flux is incorporated into the crystal, and therefore cannot contribute to droplet nucleation or to the increase in volume of existing droplets. We find that the probability for a metal adatom to attach to a forming droplet is three times larger with respect to being incorporated in the growing crystal at *F*_*T*_ (*σρ*_0_ = 3*δ*). This significant difference in incorporation probability leads to the observed fast decrease of the average growth velocity and its lack of recovery even at high *F*. When VLS growth under the droplet is considered (red curve in Fig. 2) the description of the data is substantially improved, with the mean square weighted deviation decreasing by a factor three. *R*_*T*_ still decreases with the increasing metal flux, although a sizeable decrease in the curve slope with *F*, eventually reaching a plateau at high metal fluxes, is clearly present. The VLS growth mode dominates the dynamics when the droplet coverage reaches a sizeable percentage of the surface. According to our experimental observations, the maximum coverage in our samples is χ_M_ = 0.6 (sample K, see Table I). One of fitting parameter of the model is the growth rate per unit area under the droplets (*R*_*U*_). By fitting procedure, we find that *R*_*U*_ is roughly equal, at *F*_*T*_, to *R*_*S*_. This suggests that the incorporation rate of N into the droplets and the InGaN surface at the growth temperature of 450 °C and *F*_*T*_ are equivalent.

The model predicts a recovery of the growth rate as the growth time increases, with a τ^2/3^ dependence. This is due to the increase of the area covered by the droplets whose outcome is the increase in relevance of the VLS mode. To test this expected phenomenon we grew three samples (samples G, H and I) using the same growth conditions in terms of substrate temperature, nitrogen flux and metal flux (*F* = 2.94 × 10^14^ atoms cm^−2^ s^−1^) but where we changed the growth time by a factor four (see Fig. 4b). In this sample series, a clear dependence of the average growth time is observed. The behavior of Г when τ increases follows the predictions of Eq. (7), thus confirming the model predictions that in the metal-rich zone the growth is increasingly dominated by the VLS mode in the regions covered by the droplets.

## Conclusion

The observed dynamics in the growth rate in the metal rich region is determined by the droplets. This happens in two ways. The first is related to the metal adatom attachment to the droplet that depletes the adatom density *n* thus hindering the epilayer growth. This phenomenon becomes more and more relevant as the metal flux increases, as the surface is covered by an increasing density of droplets. This leads to the observed reduction of the growth rate with the increasing metal flux. On the other side, the presence of the droplets on the surface activates the VLS crystal growth under the droplet. This relates to direct impingement of N onto the droplet. Our findings show that this growth method is faster than the normal crystal incorporation and promotes, at long times, a recovery of the growth rate in samples with high droplet coverage. It is worth mentioning that role of droplets in the growth dynamics here observed and modeled has a general validity. Because it is generated by the simultaneous presence of the competing processes of crystal growth and VLS, it therefore not restricted to case of Ga(In)N semiconductors. It is in fact applicable to growth of the wide material class of binary compounds, including As, P and Sb based semiconductors, where the excess of metal flux can cause the formation of droplets on the surface.

### Data availability

The datasets generated during and/or analysed during the current study are available from the corresponding author on reasonable request.
